# Short-term effect of ambient air pollution on outpatient visits for children in Guangzhou, China

**DOI:** 10.3389/fpubh.2023.1058368

**Published:** 2023-01-20

**Authors:** Sili Chen, Binhe Xu, Tongxing Shi, Qiaoyuan Yang

**Affiliations:** ^1^Department of Preventive Medicine, School of Public Health, Guangzhou Medical University, Guangzhou, China; ^2^Department of Clinical Medicine, Basic Medicine College, Zunyi Medical University, Zunyi, China; ^3^Department of Environmental Hygiene, Guangzhou Center for Disease Control and Prevention, Guangzhou, China; ^4^Department of Environmental Health, Institute of Public Health, Guangzhou Medical University, Guangzhou, China

**Keywords:** time-series study, outpatient, air pollution, short-term, children

## Abstract

This study examined the short-term relationship between ambient air pollutants and children's outpatient visits, and identified the effect of modifications by season. Daily recordings of air pollutants (CO, NO_2_, O_3_, SO_2_, PM_10_, and PM_2.5_) and children's outpatient visit data were collected in Guangzhou from 2015 to 2019. A generalized additive model adjusted for potential confounding was introduced to verify the association between ambient air pollution and outpatient visits for children. Subgroup analysis by season was performed to evaluate the potential effects. A total of 5,483,014 children's outpatient visits were recorded. The results showed that a 10 μg/m^3^ increase in CO, NO_2_, O_3_, SO_2_, PM_10_, and PM_2.5_ corresponded with a 0.19% (95% CI: 0.15–0.24%), 2.46% (2.00–2.92%), 0.27% (0.07–0.46%), 7.16% (4.80–9.57%), 1.16% (0.83–1.49%), and 1.35% (0.88–1.82%) increase in children's outpatient visits on the lag0 of exposure, respectively. The relationships were stronger for O_3_, PM_10_, and PM_2.5_ in the warm seasons, and for CO, NO_2_, and SO_2_ in the cool seasons. When adjusting for the co-pollutants, the effects of CO, NO_2_, and PM_10_ were robust. The results of this study indicate that six air pollutants might increase the risk of children's outpatient visits in Guangzhou, China, especially in the cool season.

## Graphical Abstract

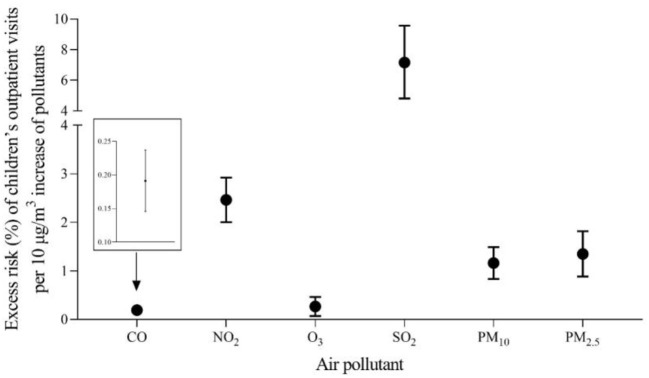

## Highlights

- The air pollutants were associated with children's outpatient visits.
- The E–R curves between pollutants and outpatient visits sometimes were positive.
- The links between air pollution and outpatient visits were different in seasons.
- Most robust associations of CO, NO_2_, and PM_10_ with children's outpatient risk.

## 1. Introduction

Ambient air pollution is a major global health issue with significant impacts worldwide, especially in developing countries. According to the Global Disease Burden, air pollution is a main cause of the global disease burden (1). Air pollution is a serious problem in China (2) due to rapid industrialization and urbanization over the past few decades. As the fourth largest environmental risk factor, air pollutants caused about 1.58 million deaths in China in 2016 (3).

Many epidemiological studies have demonstrated that exposure to air pollution, including carbon monoxide (CO), nitrogen dioxide (NO_2_), ozone (O_3_), sulfur dioxide (SO_2_), particulate matter with an aerodynamic diameter < 10 μm (PM_10_), and particulate matter with an aerodynamic diameter < 2.5 μm (PM_2.5_), can cause a range of adverse health effects. PM_10_ and PM_2.5_ are significantly associated with cardiorespiratory mortality risks, and even an increased risk of respiratory mortality (4). SO_2_, PM_10_, and PM_2.5_ are positively correlated with lung cancer mortality in Guangzhou (5). One study showed that ambient NO_2_ and PM_2.5_ exposure are significantly contacted with aggrandized all-cause non-accidental mortality (6). The adverse impacts of PM_2.5_ on cardiovascular emergency have been observed in previous studies (7, 8). Studies have also shown a positive correlation between PM_2.5_ exposure and daily medical treatment for respiratory diseases (9, 10). Moreover, SO_2_ and PM_10_ exposure are linked to increasing the risk of hospitalization for mental disorders (11). In short, previous studies have mostly reported the relationships between some of the six major air pollutants and human health effects, whereas there are few studies on the short-term relationships between six ambient air pollutants and health impacts.

Ambient outdoor and indoor air pollution caused the death of ~660,000 children in 2012 (12). Children are perhaps more sensitive than adults to the adverse health influences of air pollutants due to biological, behavioral, and environmental reasons (12). Children's exposure to ambient air pollution can have harmful and irreversible affections on organ systems because of their immature immune systems and developing lung functions (13). Compared with adults, children may inhale higher doses of air pollutants because they breathe more frequently and spend more time outdoors engaging in physical activity (14). Exposure to air pollution in infancy can cause lasting damage to cells and tissues, increase the risk of disease in children, and may have lifelong effects (15). Ambient air pollution exposure can affect child's development, so it is necessary to study the impacts of air pollutants on children's health.

Associations between children's health effects, specifically respiratory diseases, and ambient air pollutants have been well established. For example, lower respiratory diseases (16), upper respiratory tract infection (17), acute bronchitis (18), acute respiratory infections (14), pneumonia (19), and asthma (20) are significantly correlated with ambient air pollution. However, most studies have generally focused on a specific outpatient disease, and there is a lack of studies on the relationship between air pollution and children's outpatient visits for general diseases. In addition, many previous time-series studies lasted 2–4 years (18, 19, 21–23), and 5-year time-series studies have been limited.

Therefore, this study uses the time-series analysis method of quasi-Poisson generalized additive model to evaluate the acute effects of ambient air pollution (CO, NO_2_, O_3_, SO_2_, PM_10_, and PM_2.5_) on outpatient visits for children in Guangzhou, China from 2015 to 2019.

## 2. Materials and methods

### 2.1. Study location

Guangzhou (113°17′E 23°8'N), a crucial central city in China, is a comprehensive transportation hub and an international trade center (Supplementary Figure S1). Guangzhou has an oceanic subtropical monsoon climate, with a yearly average relative humidity of 77%, an average temperature of 23°C, and annual rainfall of about 1,720 mm. It has high temperatures and much rain water in the summer, and is mild and comparatively dry in winter (24). Guangzhou covers an area of 7,434.4 km^2^ and is divided into 11 municipal districts, with a resident population of 18.81 million by 2021.

### 2.2. Outpatient data

The data of children's outpatient visits were collected from January 1, 2015 to December 31, 2019 from the Guangdong Provincial Center for Disease Control and Prevention. The outpatient visits for children were obtained from six hospitals: Guangzhou Conghua District Hospital of Traditional Chinese Medicine, Guangzhou Panyu Central Hospital, Guangzhou First People's Hospital, The First Affiliated Hospital of Guangzhou Medical University, He Xian Memorial Affiliated Hospital of Southern Medical University, and the Clifford Hospital (Supplementary Figure S1).

### 2.3. Ambient air pollutants and meteorological data

During the study period, daily 24-h average concentrations of CO, NO_2_, SO_2_, PM_10_, and PM_2.5_, and maximum daily 8-h average concentration for O_3_ were obtained from the Urban Air Quality Real-Time Release Platform (https://air.cnemc.cn:18007/) of the Ministry of Ecology and Environment of the People's Republic of China. The routine average concentrations of these air pollutants were from 21 air monitoring stations spread in the urban area of Guangzhou (Supplementary Figure S1). Meteorological indicators, including the daily mean temperature and average relative humidity, were collected from the Guangdong Meteorological Service at the same time. Those two parameters were included in the model to adjust for the effects of confounding factors.

### 2.4. Statistical analyses

A children's outpatient visit is a low probability event, conforming to the Poisson distribution. A time-series quasi-Poisson generalized additive model was introduced to verify the relationships between ambient air pollution and children's outpatient visits. According to the results of previous time-series studies (25, 26), several covariates were adjusted in the model. In this main smoothing model, degrees of freedom (df) were set on the basis of previous studies (24, 27):


$$\begin{array}{l} Log\left[E\left(Y_{t}\right)\right]=\beta Z_{t}+ns\left(time,\;df=7/year\right)\\ \;+\;ns\left(temperature,\;df=6\right)\\ \;+\;ns\left(relative\;humidity,\;df=3\right)\\ \;+\;DOW_{t}+intercept, \end{array}$$

where *E* (*Y*_*t*_) is the expected number of routine children's outpatient visits on everyday t, β is the regression coefficient, *Z*_*t*_ represents the pollutant concentration at day t, *ns()* is the natural cubic curve, and *DOW*_*t*_ is the dummy variable indicating the day of the week of day t.

The potential delay effect was analyzed using various delay effect constructions. These lag models were divided into two classifications: single lag effects (lag0–lag5) and cumulative lag effects (lag01–lag05). Then, by adding a natural spline function with 4 df to the above GAM model, the exposure–response association between children's outpatient visits and ambient air pollutants were plotted. In addition, stratified analysis was carried out according to the season. The cool period was from October 1 to April 30, and the warm period was from May 1 to September 30.

The significance of a difference between two groups was examined by determining the 95% confidence interval (CI) as follows: $\left(\beta_{1}-\beta_{2}\right)\pm 1.96\sqrt{{\left(SE_{1}\right)}^{2}+{\left(SE_{2}\right)}^{2}}$, where *SE*_1_ and *SE*_2_ are their standard errors, and β_1_ and β_2_ are estimate value for every subgroup (28, 29). To test the robustness of the estimated relationships, we changed the df with 4–10 per year (30, 31). Furthermore, co-pollutant models were constructed to check the stability of the results.

We regulated all statistical analyses with R software (version 4.1.1) using the *mgcv* package. The effects are expressed as the excess risk (ER), figured out by the (relative risk – 1) × 100%, and 95% CI of children's outpatient visits per 10 μg/m^3^ increase in ambient air pollutants. *P* < 0.05 was considered statistically significant.

## 3. Results

Supplementary Figure S2 shows the time-series distributions of CO, NO_2_, O_3_, SO_2_, PM_10_, PM_2.5_, and children's outpatient visits from 1 January 2015 to 31 December 2019 in Guangzhou. A total of 5,483,014 children's outpatient visits were recorded in the six hospitals. Table 1 shows the descriptive statistics of daily ambient air pollutants, children's outpatient visits, and meteorological conditions. The daily average concentrations of CO, NO_2_, O_3_, SO_2_, PM_10_, and PM_2.5_ were 892.8, 46.8, 90.1, 10.4, 55.3, and 34.5 μg/m^3^, respectively. These values for CO, NO_2_, SO_2_, PM_10_, and PM_2.5_ were about 0.2, 1.2, 0.2, 0.8, and 1.0 times the secondary standard limits of GB 3095-2012 set by China (4,000.0, 40.0, 60.0, 70.0, and 35.0 μg/m^3^ annually), and 0.2, 4.7, 0.3, 3.7, and 6.9 times the ambient air quality standards in World Health Organization (WHO) (4,000.0, 10.0, 40.0, 15.0, and 5 μg/m^3^ annually), respectively. O_3_ concentrations on 193 and 699 days exceeded the daily criteria set by China (160 μg/m^3^) and the WHO (100 μg/m^3^), respectively. The daily average value of relative humidity was 80.3%, and the annual mean temperature was 22.3°C in Guangzhou.

**Table 1:** Daily air pollutants, meteorological data, and children's outpatient visits during the study.

	**Mean**	**SD**	**MIN**	**P25**	**P50**	**P75**	**MAX**
**Air pollution concentration (**μ**g/m**^3^**)**							
CO	892.8	224.0	400.0	700.0	900.0	1,000.0	2,100.0
NO_2_	46.8	19.1	8.0	34.0	42.5	55.0	168.0
O_3_	90.1	52.3	0.0	49.0	84.0	122.0	287.0
SO_2_	10.4	4.4	3.0	7.0	10.0	13.0	37.0
PM_10_	55.3	27.3	9.0	36.0	48.0	70.8	212.0
PM_2.5_	34.5	19.1	5.0	21.0	30.0	44.0	155.0
**Meteorological measures**							
Humidity (%)	80.3	10.3	31.0	75.0	82.0	88.0	100.0
Temperature (°C)	22.3	5.9	3.6	17.9	23.5	27.3	31.2
No. of daily children's outpatient visits	3,003	632	221	2,632	3,014	3,390	5,225
**Season (N)**							
Cool	3,029	663	221	2,694	3,074	3,416	5,225
Warm	2,966	584	1,465	2,577	2,930	3,354	4,816

Table 2 displays the Spearman correlation coefficients among ambient air pollution and meteorological elements in Guangzhou, China. The six air pollutants were positively correlated with each other, except O_3_ and CO, and negatively correlated with temperature, except O_3_. Moreover, relative humidity was negatively correlated with O_3_, SO_2_, PM_10_, and PM_2.5_, and positively correlated with temperature, CO, and NO_2_. Significant correlations were observed between the exposure variables, except between SO_2_ and temperature and between NO_2_ and relative humidity.

**Table 2:** Spearman's correlations between daily average air pollution concentrations and meteorological factors during the study period.

	**CO**	**NO_2_**	**O_3_**	**SO_2_**	**PM_10_**	**PM_2.5_**	**Humidity**
NO_2_	0.57^**^						
O_3_	−0.17^**^	0.10^**^					
SO_2_	0.39^**^	0.55^**^	0.29^**^				
PM_10_	0.52^**^	0.75^**^	0.37^**^	0.67^**^			
PM_2.5_	0.59^**^	0.74^**^	0.30^**^	0.64^**^	0.96^**^		
Humidity	0.09^**^	0.03	−0.45^**^	−0.35^**^	−0.34^**^	−0.30^**^	
Temperature	−0.39^**^	−0.34^**^	0.43^**^	−0.04	−0.23^**^	−0.31^**^	0.16^**^

** P < 0.01.

**Figure 1:**
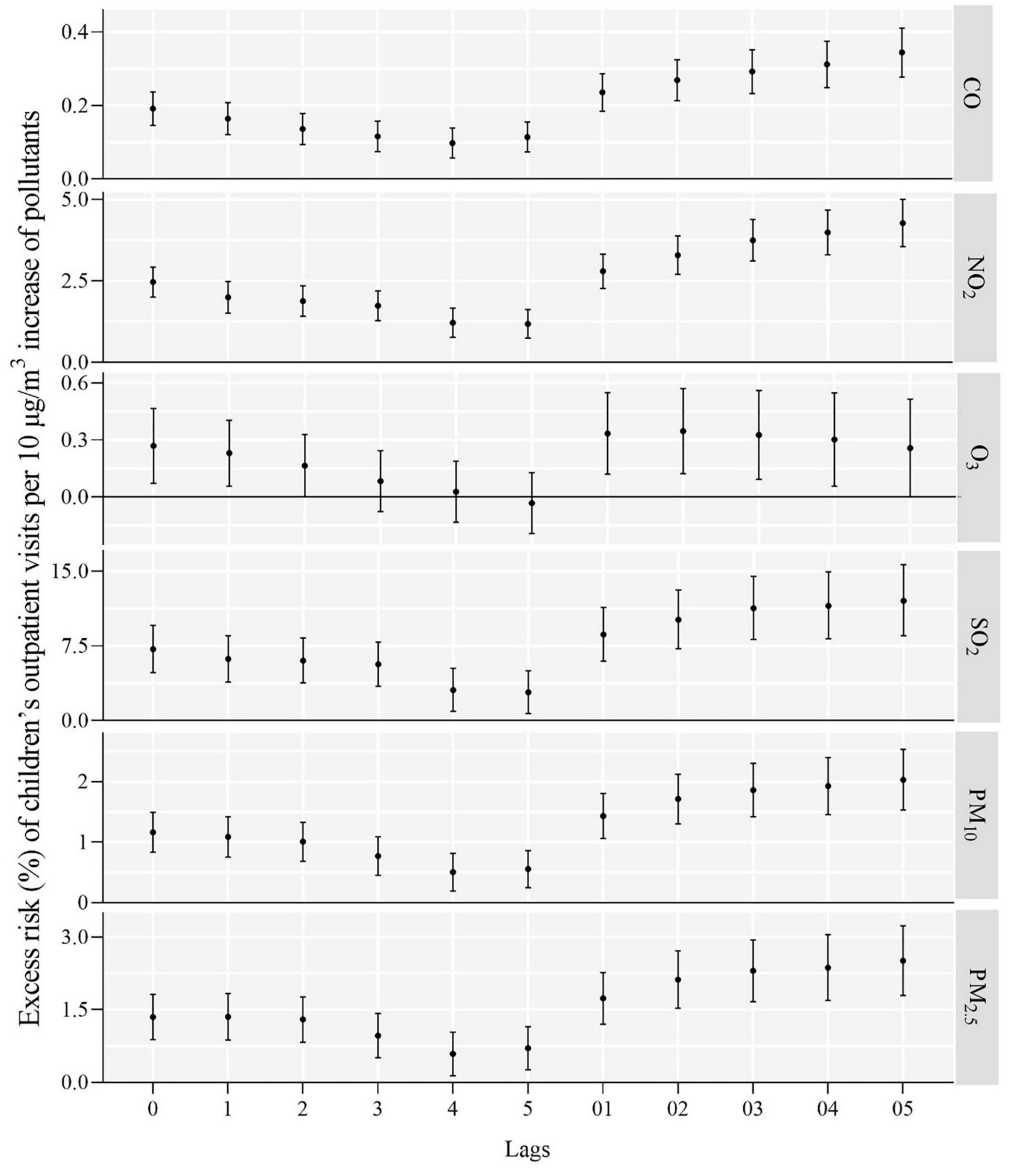
Excess risk (%) and 95% confidence intervals of children's outpatient visits a 10 μg/m^3^ increase in various ambient air pollutant concentrations along different lag days.

As shown in Figure 1, a 10 μg/m^3^ increase in CO, NO_2_, SO_2_, PM_10_, and PM_2.5_ was connected with excess risk of outpatient visits for children after adjusting for relative humidity and temperature. The estimated effects on risk of air pollutant concentrations were tested using models with different single lag days. The relationships between air pollution and children's outpatient visits were statistically significant, except for O_3_ in the model with lag3–5 in the single-pollutant. According to the model fit statistics, this means that each 10 μg/m^3^ increase in ambient CO, NO_2_, SO_2_, PM_10_, and PM_2.5_ corresponded to a 0.19% (95% confidence interval:0.15–0.24%), 2.46% (2.00–2.92%), 0.27% (0.07–0.46%), 7.16% (4.80–9.57%), 1.16% (0.83–1.49%), and 1.35% (0.88–1.82%) increase in children's outpatient visits on the day (lag0) of exposure, respectively. The cumulative lag days, like single lag days, ranging from lag01 to lag05. Overall, the influences of six air pollutants on the cumulative lag days were stronger than on the single days. The maximum cumulative effects were observed on lag02 for O_3_, 0.35% (0.12–0.57%), and lag05 for CO, 0.34% (0.28–0.41%), NO_2_, 4.27% (3.55–5.00%), SO_2_, 12.01% (8.50–15.64%), PM_10_, 2.03% (1.53–2.53%), and PM_2.5_, 2.51% (1.79–3.23%).

**Figure 2:**
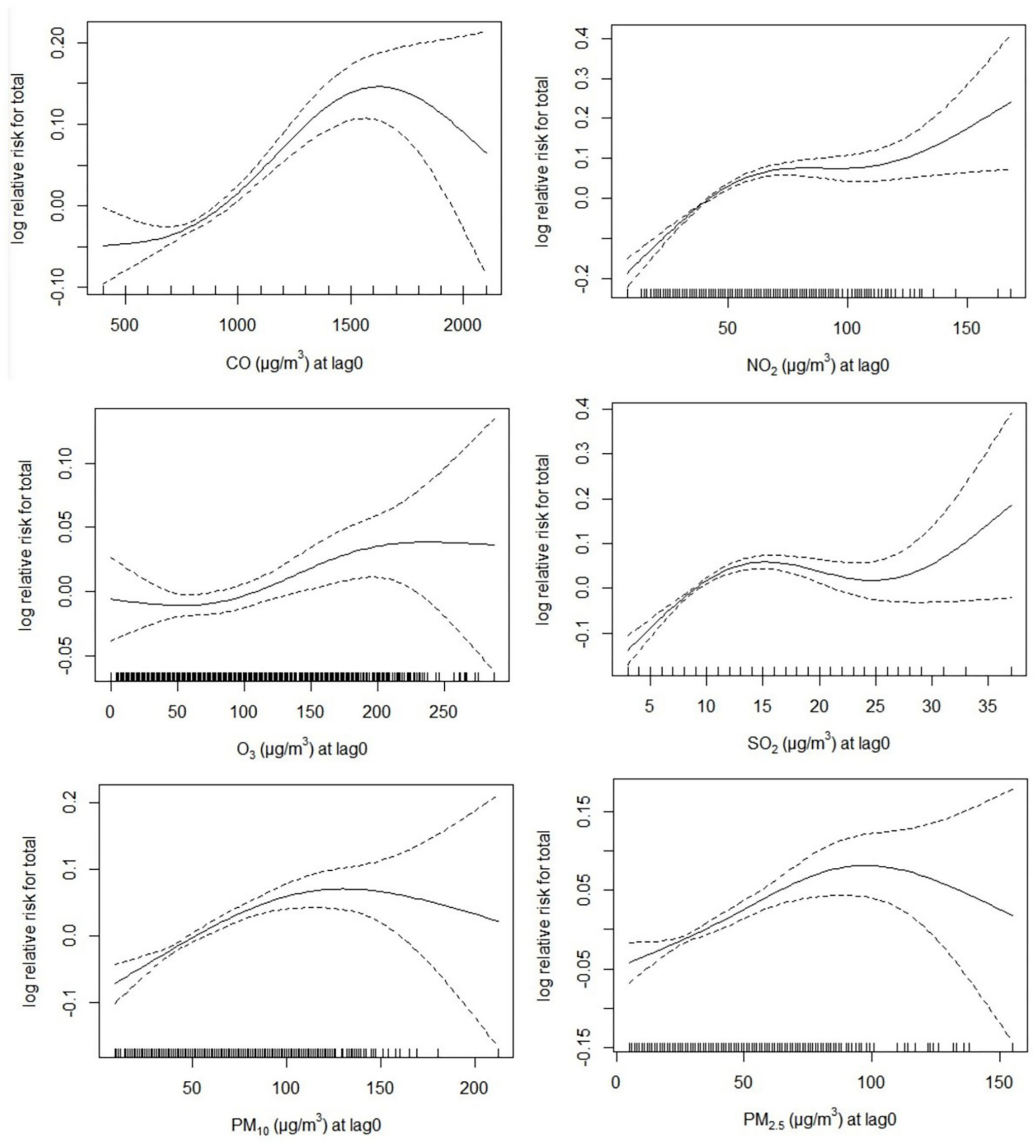
The exposure–response curves of the associations of different air pollution with the risk of children's outpatient visits in single-pollutant model. The black line represents the average relative risk of the pollutant concentration, and the dashed lines are the 95% confidence interval of the risk estimates.

The exposure–response curves of the correlations between ambient air pollution and children's outpatient visits are given in Figure 2. The curves of the ambient air pollution were obviously positive in the meaningful exposure range. The exposure–response curves of CO, PM_10_, and PM_2.5_ displayed a sharp pitch at concentrations < 1,500 μg/m^3^, < 100 μg/m^3^, and < 100 μg/m^3^ and then a decrease. The curves of O_3_ were nearly S-shaped, sharply rising at concentrations ≥100 μg/m^3^ and flattening for concentrations ≥200 μg/m^3^. The exposure–response curves of NO_2_ and SO_2_ showed steep hill at concentrations < 50 and < 15 μg/m^3^, rapidly ascended at concentrations ≥100 and ≥25 μg/m^3^, and became flattened at mid-range concentrations.

Table 3 shows the estimated ER of children's outpatient visits and 95% CI by season breakdown of the concentrations of ambient air pollution. Season stratification revealed significant differences in the links between ambient air pollutants and the risk of children's outpatient visits. The associations with O_3_, PM_10_, and PM_2.5_ were more prominent in warm than in cool seasons, and the links with CO, NO_2_, and SO_2_ were much stronger in cool seasons. Specifically, relationships between O_3_ and children's outpatient visits were non-significant in the cool seasons.

**Table 3:** Estimated ER and 95% CI of children's outpatient visits a 10 μg/m^3^ increase in the concentrations of air pollution stratified by season.

**Pollutant**	**Whole**	**Season**	
		**Cool**	**Warm**
CO	0.19 (0.15 to 0.24)	0.16 (0.10 to 0.21)^*^	0.08 (0.01 to 0.16)^*^
NO_2_	2.46 (2.00 to 2.92)	2.03 (1.52 to 2.55)^*^	1.82 (0.86 to 2.80)^*^
O_3_	0.27 (0.07 to 0.46)	0.15 (−0.14 to 0.45)^*^	0.29 (0.08 to 0.50)^*^
SO_2_	7.16 (4.80 to 9.57)	5.67 (2.86 to 8.57)^*^	4.82 (0.95 to 8.85)^*^
PM_10_	1.16 (0.83 to 1.49)	0.74 (0.36 to 1.11)^*^	1.00 (0.37 to 1.63)^*^
PM_2.5_	1.35 (0.88 to 1.82)	0.82 (0.29 to 1.36)^*^	1.30 (0.41 to 2.20)^*^

* The difference between groups was statistically significant.

**Figure 3:**
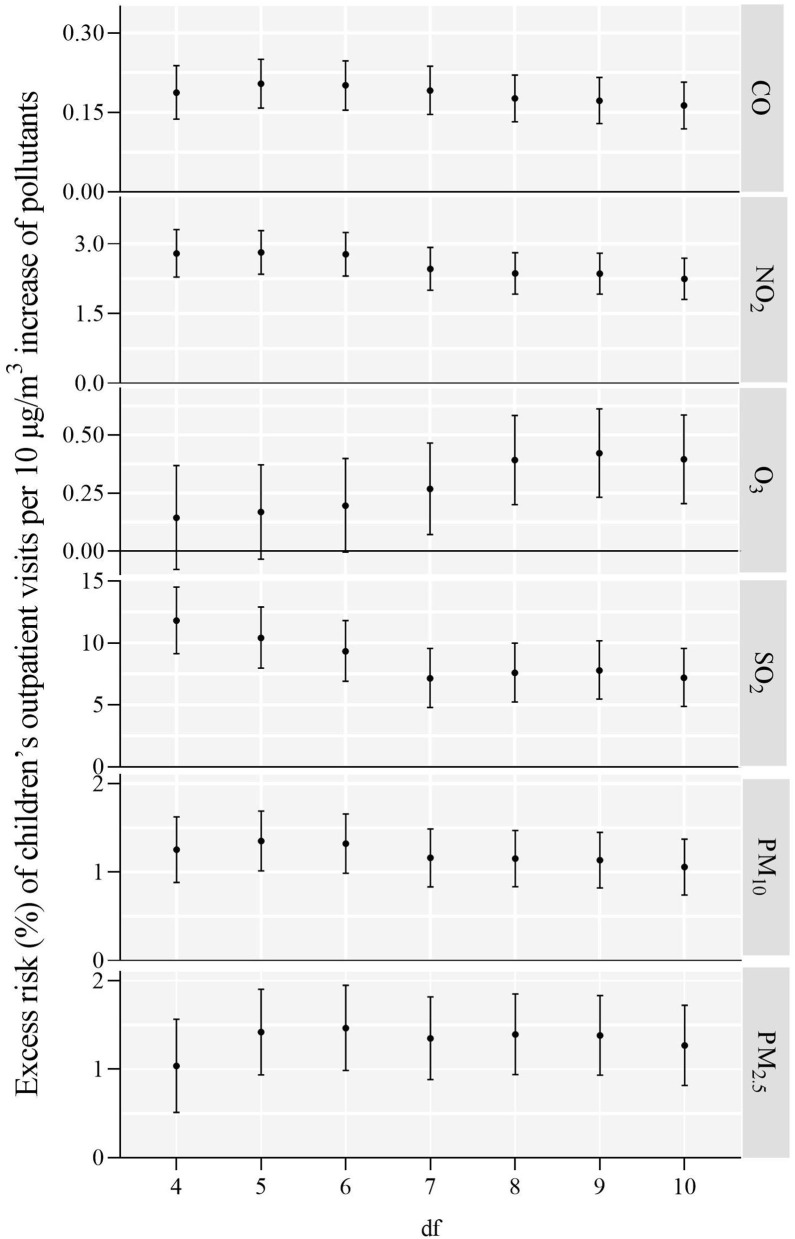
ER (%) and 95% CI of children's outpatient visits a 10 μg/m^3^ increase in air pollution concentrations on the day of exposure.

In the sensitivity analysis, when we use df to adjust the smoothness of time from 5 to 9, the results did not change substantially, except for O_3_ (Figure 3). Table 4 establishes the relationships between all ambient pollutants and children's outpatient visits in two-pollutant models, as assessed by another sensitivity analysis. In the two-pollutants analysis, the Spearman's correlation coefficients < 0.7 were introduced into the co-pollutant models. The correlations of CO, NO_2_, and PM_10_ in the risk of children's outpatient visits remained robust in the co-pollutant models. However, after adjusting for four air pollutants, the effects of O_3_ became non-significant, while the adjustment for NO_2_ yielded significant results. When adjusting for NO_2_ and PM_10_, the influences of SO_2_ decreased and became not significant, respectively. After adjusting for CO, the effects of PM_2.5_ decreased and became non-significant. Together, these results indicate that CO, NO_2_, and PM_10_ may play more alone role in children's outpatient risk.

**Table 4:** ER (%) and 95% CI of children's outpatient visits in co-pollutant models.

**Two-pollutant models**		**Estimates**
CO	–	0.19 (0.15 to 0.24)^*^
	+NO_2_	0.08 (0.02 to 0.13)^*^
	+O_3_	0.19 (0.14 to 0.23)^*^
	+SO_2_	0.16 (0.11 to 0.21)^*^
	+PM_10_	0.15 (0.09 to 0.21)^*^
	+PM_2.5_	0.18 (0.12 to 0.24)^*^
NO_2_	–	2.46 (2.00 to 2.92)^*^
	+CO	2.01 (1.46 to 2.56)^*^
	+O_3_	2.73 (2.22 to 3.25)^*^
	+SO_2_	2.85 (2.21 to 3.50)^*^
O_3_	–	0.27 (0.07 to 0.46)^*^
	+CO	0.06 (−0.14 to 0.26)
	+NO_2_	−0.25 (−0.46 to −0.04)^*^
	+SO_2_	0.04 (−0.17 to 0.25)
	+PM_10_	−0.10 (−0.33 to 0.12)
	+PM_2.5_	−0.01 (−0.23 to 0.22)
SO_2_	–	7.16 (4.80 to 9.57)^*^
	+CO	3.51 (0.98 to 6.10)^*^
	+NO_2_	−2.64 (−5.59 to 0.39)
	+O_3_	6.98 (4.43 to 9.59)^*^
	+PM_10_	2.53 (−0.74 to 5.91)
	+PM_2.5_	4.82 (1.58 to 8.16)^*^
PM_10_	–	1.16 (0.83 to 1.49)^*^
	+CO	0.50 (0.09 to 0.92)^*^
	+O_3_	1.25 (0.87 to 1.63)^*^
	+SO_2_	0.89 (0.41 to 1.37)^*^
PM_2.5_	–	1.35 (0.88 to 1.82)^*^
	+CO	0.17 (−0.43 to 0.77)
	+O_3_	1.36 (0.83 to 1.89)^*^
	+SO_2_	0.65 (0 to 1.31)^*^

* P < 0.05.

## 4. Discussion

We conducted a time-series study to assess the short-term relationships between ambient air pollution and children's outpatient visits and observed significant relationships. Exposure–response relationships between six ambient air pollutants and the risk of children's outpatient visits were positive in the meaningful exposure range. The associations with O_3_, PM_10_, and PM_2.5_ were more prominent in warm than in cool seasons, and the links with CO, NO_2_, and SO_2_ were stronger in cool than in warm seasons. The correlations of O_3_, SO_2_, and PM_2.5_ with children's outpatient visits were likely affected by other air pollutants, whereas CO, NO_2_, and PM_10_ appeared to play more alone role in the risk of children's outpatient visits. This analysis provides the latest evidence to establish the relationships between air pollutants and harmful health effects.

Significant relationships between CO, NO_2_, O_3_, SO_2_, PM_10_, PM_2.5_, and children's outpatient visits were found in this research, which was commonly consistent with previous time-series studies (22, 32–34). Each 10 μg/m^3^ increase of O_3_ and PM_2.5_ corresponded to 0.27% (0.07–0.46%) and 1.35% (0.88–1.82%) increments in the risk of children's outpatient visits in the single-pollutant model, respectively, but these were not significant in the co-pollutant models, match with previous studies (31, 35). In the co-pollution model, the adverse effects of O_3_ disappeared or even reversed may be explained by the relative instability of ozone (36). A weak but significant relationship between CO and children's outpatient visits was found, in line with a previous study (30, 37). In the analysis of the two pollutant models, the associations with NO_2_ and PM_10_ remained robust, which may reveal that NO_2_ and PM_10_ had more independent effects on children's outpatient visits. Previous studies have indicated that the level of significance did not increase when SO_2_ be analyzed in co-pollutant model (38, 39).

Although exposure–response relationships may change due to a variety of limitations, including climatic characteristics, geographical location, air pollution mixtures, and population sensitivity (11, 31), the results still have reminders for human health assessment. In this research, a linear and limitation association between CO, PM_10_, and PM_2.5_ the risk of children's outpatient visits were identified within a certain range, whereas a linear and not threshold association between O_3_, NO_2_, SO_2_ and children's outpatient visits were confirmed. The curve for NO_2_ tended to plateau at mid-range concentrations. The S-shaped curve for O_3_ tended to plateau at high concentrations. As a result, ambient air pollutant concentrations should be constantly reduced to protect human health and reduce the risks of outpatient visits among children.

The differences in the effects of CO, NO_2_, O_3_, SO_2_, PM_10_, and PM_2.5_ on the risk of children's outpatient visits between cold and warm seasons were statistically significant. The influences of PM_10_ and PM_2.5_ on this risk were significantly stronger in warm seasons, consistent with previous studies (31, 40). The possible reason may be that children spend more time outdoors in warm than in cold seasons. So their exposure dose perhaps lower during the cold seasons (31). The relationships of SO_2_ and NO_2_ with children's outpatient visits were stronger in the cool season. This finding was in agreement with several studies (41, 42) but contradictory to others (33, 35). The specific reason for these differences between warm and cool seasons must be clarified in the future.

The observed influences of ambient air pollution on children's outpatient visits are biologically plausible. NO_2_ and SO_2_ can augment the permeability of airway mucosa and increase allergic diseases (43). NO_2_ can also induce airway inflammation and may restrict the smaller airways and terminal bronchioles (44). PM_2.5_ and PM_10_ can be deeply inhaled into the lungs (45), causing various inflammatory reactions (46–48), and local inflammation of alveoli can further develop into systemic inflammatory (49). O_3_ affects airway inflammation in children by increasing the levels of cationic proteins associated with leukocytes and eosinophils (50). PM_2.5_, SO_2_, and NO_2_ can increase airway oxidative stress and reduce small airway function (51).

This study has a few limitations. First, we used in average pollutant concentrations from fixed-site monitors rather than individual monitoring data, which might have resulted in exposure errors (52). Second, although we used data on children's outpatient visits from six hospitals in the study city, selection bias might have existed. Third, due to the limitations of the collected children's outpatient records, we can only get the number of children's outpatients per day, without studying other stratification analyses and finding multiple visits during a course of illness (26). Fourth, none of the hospitals is a children's hospital which has the largest number of children's outpatient visits in the city. In addition, the matching between environmental exposure and illness is not enough because of the lack of the children's residential address and monitoring data from the nearest monitoring stations according to the location of hospitals, which is also the inadequacy of this study. Finally, we did not include confounding factors at the personal level (e.g., lifestyles and indoor pollution exposure) (53), which might affect the relationships between ambient air pollution and individual vulnerability.

## 5. Conclusions

This time-series analysis suggests that ambient air pollution, especially CO, NO_2_, and PM_10_, can significantly increase the risk of children's outpatient visits in Guangzhou, China. The relationships were stronger for CO, NO_2_, and SO_2_ in the cool seasons, and for O_3_, PM_10_, and PM_2.5_ in the warm seasons. The findings of this study suggest that persistent efforts to reduce air pollution levels in Guangzhou would have health benefits, resulting in a decrease in children's outpatient visits. These results can only be generalized to cities with similar populations, societies, and environments.

## Data availability statement

The original contributions presented in the study are included in the article/Supplementary material, further inquiries can be directed to the corresponding author.

## Author contributions

QY designed the study and supervised the research, including funding, text review, and overall quality assurance and control. SC helped with the formulation of research methods, software analysis, and wrote the original draft of the text. BX helped with the investigation and review of the data and assisted in the preparation of the original draft of the text. TS assisted in the implementation of research, data management, investigation, and supervision. All authors contributed to the article and approved the submitted version.

## Supplementary material

Supplementary Figure S1 The locations of 21 ambient air monitoring stations and 6 hospitals in Guangzhou between 1 January 2015 and 31 December 2019.

Supplementary Figure S2 The time-series distributions of CO, NO_2_, O_3_, SO_2_, PM_10_, PM_2.5_ and outpatient visits from 1 January 2015 to 31 December 2019 in Guangzhou.
