# Supplementary figures and images for: Short-term effect of ambient air pollution on outpatient visits for children in Guangzhou, China
Source: Front Public Health. 2023 Jan 20;11:1058368. doi: 10.3389/fpubh.2023.1058368 (PMC9895100; doi:10.3389/fpubh.2023.1058368)

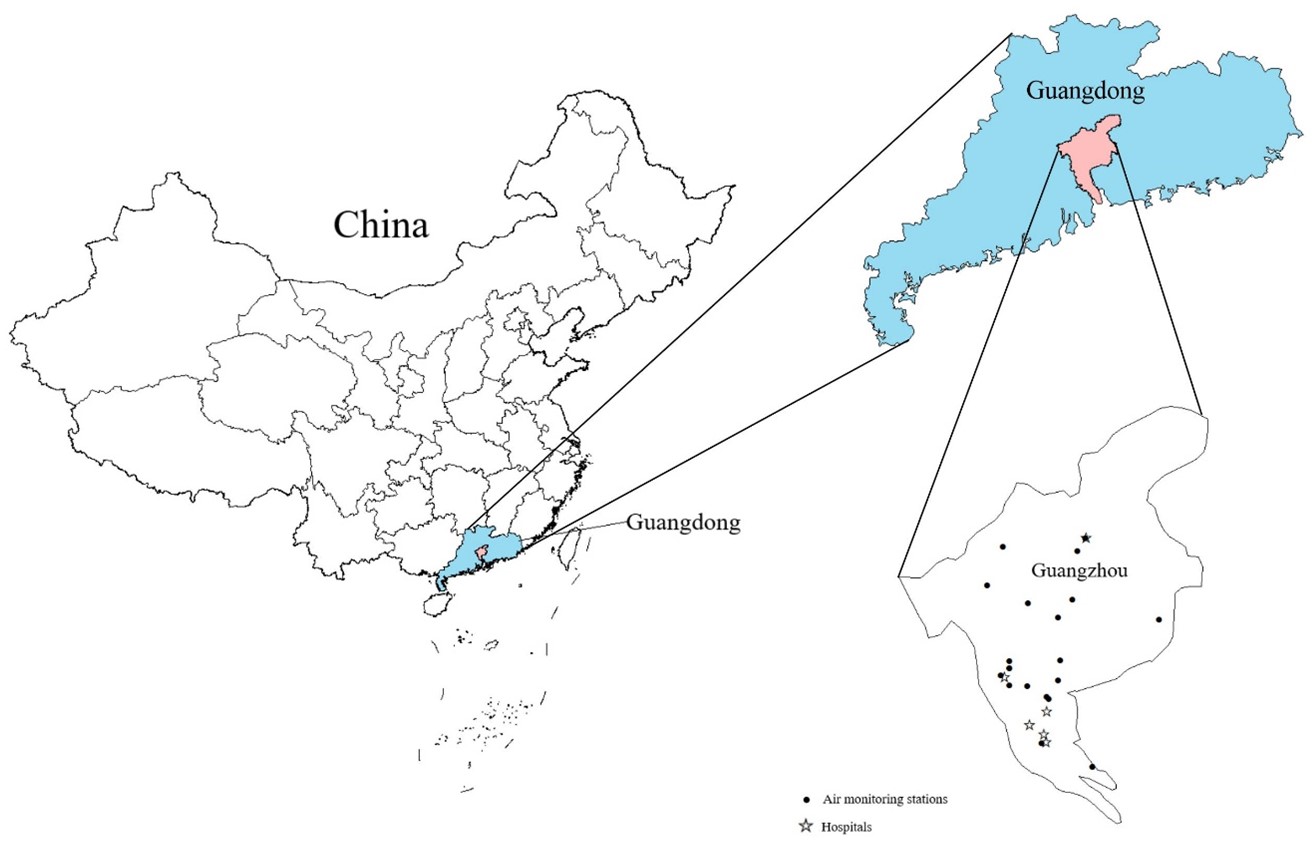

Supplement: Supplementary Figure S1 — The locations of 21 ambient air monitoring stations and 6 hospitals in Guangzhou between 1 January 2015 and 31 December 2019. [file Image_1.JPEG]

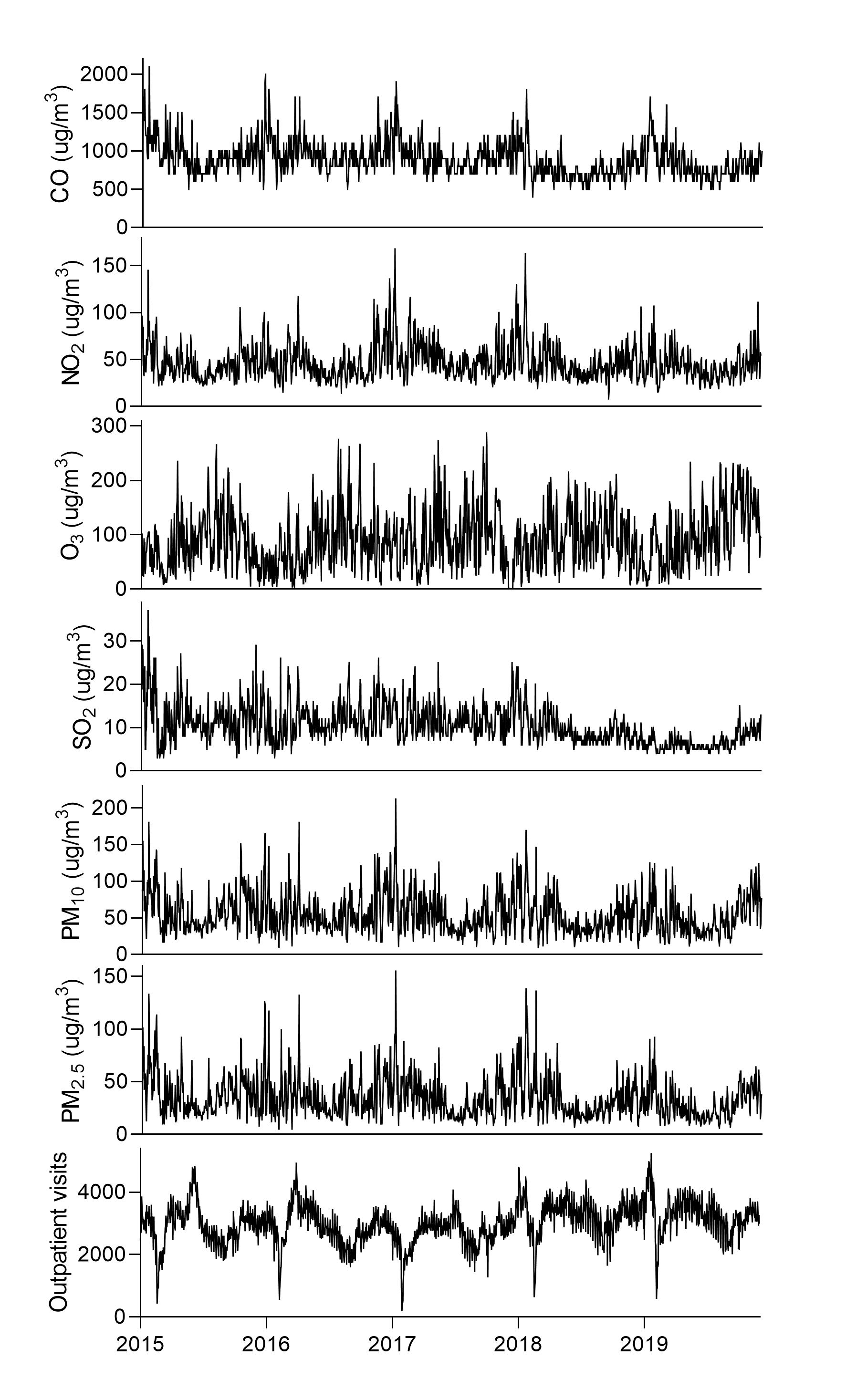

Supplement: Supplementary Figure S2 — The time-series distributions of CO, NO2, O3, SO2, PM10, PM2.5 and outpatient visits from 1 January 2015 to 31 December 2019 in Guangzhou. [file Image_2.JPEG]
